# Application of density gradient for the isolation of the fecal microbial stool component and the potential use thereof

**DOI:** 10.1038/srep16807

**Published:** 2015-11-19

**Authors:** Arancha Hevia, Susana Delgado, Abelardo Margolles, Borja Sánchez

**Affiliations:** 1 Department of Microbiology and Biochemistry of Dairy Products, Dairy Research Institute (IPLA-CSIC), Paseo Río Linares s/n, 33300 Villaviciosa, Asturias, Spain

## Abstract

The idea of considering the gut microbiota as a virtual human organ has led to the concept of fecal microbiota transplantation (FMT), which has recently been extremely successful in the treatment of cases of recurrent *Clostridium difficile* infection. Administration of safe, viable, and representative fecal microbiota is crucial for FMT. To our knowledge, suitable techniques and systematic conditions for separating the fecal microbiota from stool samples have not been thoroughly investigated. In this work we show the potential to separate stool microorganisms from the rest of fecal material using a procedure with a Nycodenz® density gradient, yielding 10^10^ viable bacteria per two grams of feces. This procedure did not affect the original microbiota composition in terms of viability, distribution and proportions, as assessed by a phylogenetic metagenomic approach. Obtaining the fecal microbiota by concentration and separation of the microorganisms from the rest of the stool components would allow the standardization of its recovery and its long-term preservation. FMT or similar microbiota restoration therapies could be used for the treatment of several disorders, or even for aesthetic purposes, so the method described in our work may contribute to the setting of the basis for the development of safe and standardized products.

The human gastrointestinal tract (GI) is a complex ecosystem in which the resident microbiota and nutrients continuously interact with host cells^1^. Gut microbiota is composed of trillions of bacteria, outnumbering the eukaryotic cells of our body in one order of magnitude^2^, and the idea of considering our intestinal microbiota as a virtual organ is gaining popularity among the scientific community^3^. Genes provided by our gut microbiota are denominated ‘gut microbiome’, but sometimes the term ‘human microbiome’ (theoretically the gene complement of all the microbes inhabiting our body) is used as a synonym^4^. Accounting for nearly 10,000,000 unique genes, notably greater than the “modest” number of 21,000 human genes^5^,^6^, our gut microbiota complement metabolic attributes that are absent in our organism, including the ability to take advantage of otherwise non-metabolizable nutrients, the production of short-chain fatty acids or vitamins, and many others^7^. On the contrary, the human host provides microbiota with nutrients *i.e*., our GI is a kind of microbial garden where each individual farms its own beneficial microbes.

During the last few years the great advance of high-throughput sequencing technologies and their application in the study of gut microbial communities, is providing growing evidence that gut microbiota has an important impact in the successful maturing of our immune system and in several facets of human physiology, which may include the triggering, progression and establishment of several diseases. Not only are the gut microbiota profiles affected by diet^8^,^9^, age^10^, or geography^11^, alteration of our intestinal microorganism composition has been linked to some gut and autoimmune disorders such as obesity^12^, metabolic syndrome^13^, rheumatoid arthritis^14^, type-1 diabetes^15^, inflammatory bowel diseases^16^, and systemic lupus erythematosus (SLE)^17^.

If we agree that the human microbiota is one more of our organs, the concept of fecal microbiota transplantation (FMT) will quickly arise. In scientific literature, FMT was first described in the late 1950s^18^, and can be defined as the ‘delivering of processed stools from a healthy individual to the gut of a sick person through enema, colonoscopy or other means’^19^. Notably, FMT had an impressive efficacy (more than 90% success in some cases) in the displacement of recurrent *Clostridium difficile* infection from the intestine of affected individuals who were not responding to antibiotic therapy, and in the re-establishment of a balanced gut microbiota^20^. Very recently, FMT was successfully applied to treat antibiotic induced colitis^21^. Generalization of unregulated FMT in certain populations led the US Food and Drug Administration (FDA) to strictly regulate faeces as a biological drug^22^.

These properties of FMT on human gut health have had a high impact in society, including thousands of entries on well-known social networks and blogs, as well as the creation of foundations and other non-profit organizations dedicated to the promotion of FMT application (*e.g.* http://thefecaltransplantfoundation.org/, http://thepowerofpoop.com/, http://www.openbiome.org/). Current methodology for FMT includes processing of fresh donor feces in the same day. However, some protocols have been published in order to separate the intestinal microbiota from the rest of fecal material by microfiltration, allowing for instance its storage^23^. Following this protocol, the intestinal microbiota are firstly microfiltered in the presence of a cryoprotectant and then frozen at −80 °C. This microbial preparation has been shown as effective as a fresh feces preparation for the displacement of *Clostridium difficile*, as evidenced by 16S rRNA gene profiling^24^.

Potential applications of FMT, other than recurrent *C. difficile* infections, are numerous but deserve studies on the normalization and standardization of what is the healthy fecal microbiota. Firstly, extracting the microbiota and its separation from the rest of the stool material under controlled conditions could serve to avoid the unappealing nature of feces. Secondly, this could allow the long-term preservation of fecal microbiota, allowing its propagation in bioreactors, even many years after its extraction. In addition, it will facilitate tasks such as stool screening for viruses (HIV, hepatitis and others), parasites and other undesirable microorganisms.

In this work, we describe the application of a fecal microbiota separation procedure by the use of a density gradient. Using 16S rDNA metagenomic profiling we confirmed that the overall microbial community structure remained unaltered after being separated from the stools. Potential applications for this method for the long-term preservation of the intestinal microbiota are also discussed.

## Materials and Methods

### Ethical Statement

Ethics approval for this study was obtained within the framework of the project AGL2010–14952, from the Spanish Ministry of Economy and Competitiveness (“Towards a better understanding of gut microbiota functionality in some immune disorders”). Final approval was obtained from the Bioethics Committee of CSIC (Consejo Superior de Investigaciones Científicas) and from the Regional Ethics Committee for Clinical Research (Servicio de Salud del Principado de Asturias) in compliance with the Declaration of Helsinki. All determinations were performed with fully informed written consent from all participants involved in the study.

### Study subjects

Stool samples from this study were obtained from five Systemic Lupus Erythematosus (SLE) patients and three healthy controls, selected from a previous study in which the gut microbiota dysbiosis associated with SLE was described^17^. Detailed clinical and nutritional data from those participants can be retrieved in the above mentioned study. SLE patients not used antibiotics, glucocorticoids, immunosuppressive drugs, monoclonal antibodies or other immunotherapies during the 6 months prior to sample collection.

### Stool samples, microbiota separation and DNA extraction

**Figure 1:**
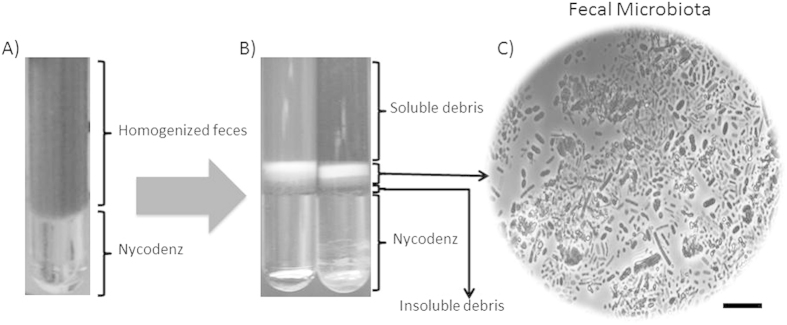
Workflow of the experimental setup used in this work. (**A**) Diluted homogenized fecal samples were loaded on top of a Nycodenz® solution, as described in the material and methods section. (**B**) After centrifugation four layers were formed. Examination of the layer content in a phase-contrast microscope allowed us to determine the presence of one layer, corresponding to the fecal microbiota, between two layers containing soluble (upper) and insoluble (lower) fecal debris all above the Nycodenz. (**C**) Light photography of the microbiota layer, showing a high diversity of microbial sizes and shapes. Bar, 10 μm.

A part of each stool sample was submitted to a density gradient in order to separate the microbiota from the rest of the fecal material, according to the method of Courtois and colleagues with some modifications^25^. Two grams of feces were homogenized in 18 mL of sterile NaCl 0.9% (w/v), in a laboratory paddle blender (Stomacher Lab Blender 400, Seward Ltd. UK) for 1 min. A solution of Nycodenz® 80% (w/v) (PROGEN Biotechnik GmbH, Heidelberg, Denmark) was prepared in ultrapure water, and sterilized at 121 °C for 15 min. A volume of 10.5 mL of the diluted, homogenized fecal sample was placed on top of 3.5 mL of the Nycodenz® solution, and centrifuged for 40 min at 4 °C (10,000 × g, TST41.14 rotor, Kontron, Milan, Italy). The upper phase, containing soluble debris, was discarded after the centrifugation step, and the layers corresponding to the microbiota extracted with 10.5 mL of PBS (Fig. 1) were collected. Cellular suspensions were kept on ice for 5 minutes, in order to allow non-soluble debris to precipitate, were then washed twice, and stored in aliquots of 1 mL, at −80 °C, until DNA extraction was performed. In all the cases, DNA directly from homogenized stool samples, or from the corresponding separated microbiota fractions was extracted using the QIAamp DNA Stool Mini kit (Qiagen Ltd., Strasse, Germany), as described in a previous work^26^.

### Efficiency and yield of the microbiota separation procedure

In order to evaluate the yield of the microbiota extraction and establish the viability of the microbiota recovered, three additional fecal samples from three healthy donors were analyzed by flow cytometry before and after density gradient extraction. For enumeration of bacteria the samples were measured using a flow cytometer (Cytomics FC500, Beckman-Coulter Inc., Miami, Florida, USA) with the Bacteria counting kit (Invitrogen^TM^, Life Technologies, Thermo Fisher Scientific, Waltham, MA). The absolute counting values in the samples were determined taking a minimum of 2,000 and a maximum of 10,000 fluorescent standard beads, and according to the analysis of the areas corresponding to beads: alive bacteria (stained with Syto9) and dead bacteria (stained with propidium iodide, Sigma, St. Louis, MO). The trigger signal was established at side scatter (SSC) detector (as recommended by the Bacteria counting kit, Invitrogen) and fluorescence signals were collected at FL1 detector (510–550 nm) for Syto9 and FL4 detector (660–700 nm) for propidium iodide. Microfiltered PBS was used as negative control. Additionally, as control of dead microbiota, we treated an aliquot of the fecal samples at 98 °C for 10 min plus 15 min under UV light exposition. The viability of microbiota in each sample was calculated as the percentage of live bacteria within the fecal microbiota before and after the Nycodenz® extraction procedure. The absolute number of bacteria was calculated using the fluorescent beads as internal standard in each sample, following the supplier’s recommendations for ratiometric counting. Relative concentrations were expressed as the absolute number of bacteria in relation to grams of total dry fecal matter, which was determined according to FIL-IDF standards^27^,^28^.

### 16 sRNA gene profiling analysis.

Partial 16S rRNA gene amplicons were obtained with primers Probio_Uni and Probio_Rev (targeting the V3 and V4 region) by PCR as described in previous works of our research group^17^,^26^. Sequence libraries using the Ion Sequencing 200 kit (Life Technologies, Thermo Fisher Scientific, Waltham, MA), were prepared from the purified PCR products and sequenced in an Ion Torrent PGM system at the GenProbio Ltd facilities (http://www.genprobio.com). After sequencing, specific sequence read groups such as low quality and polyclonal sequences were removed by the PGM software. Sequences matching the PGM 3′ adaptor were also automatically trimmed. All PGM quality-approved, trimmed and filtered data were exported as .sff files.

The .sff files were processed using QIIME 1.7.0 with the scripts and procedures described in previous works^26^,^29^. Only sequence reads with a length of between 150 and 200 bp, as well as with a mean sequence quality score higher than 25 were retained as part of the quality control. Sequences were trimmed at the first base if a low quality rolling 10 bp window was found, and other sequences such as homopolymers (>7 bp), or sequences with mismatched primers were omitted. In order to calculate downstream diversity measures (alpha and beta diversity indices, Unifrac analysis), 16S rRNA Operational Taxonomic Units (OTUs) were defined at ≥97% sequence homology and chimeric sequences were removed using Chimera Slayer^30^. All reads were classified to the lowest possible taxonomic rank using QIIME and a reference dataset from GreenGenes (version 13.5, May 2013, http://greengenes.secondgenome.com), but in general family level was the lowest taxonomic unit considered throughout the study. OTUs were assigned using uclust by using the script pick_de_novo_otus.py provided with QIIME and exported in BIOM format for downstream analyses^31^. Different alpha diversity metrics (Chao, Observed Species, Shannon and Simpson) were calculated from the BIOM formatted tables using the alpha_diversity.py script provided by QIIME.

### Statistical analysis

16S rRNA gene profiles before or after density gradient extraction were evaluated at four taxonomic levels (Phylum, Class, Order and Family). Samples were ordered according to their microbial profiles using three different and unsupervised multivariate analysis, Principal Component Analysis (PCA), Principal Coordinate Analysis (PCO) and Correspondence Analysis (CA), implemented in the software PAST v3.0^32^. After ordering, samples were classified according to the sample type used for DNA extraction (feces versus microbiota separated on Nycodenz® density gradient). Different statistical tests were conducted on the multivariate data, including One-way ANOSIM and One-way PERMANOVA, each one with 9,999 permutations. In order to assess differences in single taxonomic groups, OTU tables in BIOM format were collapsed at the four taxonomic levels, exported in tab-delimited text format and analyzed using STAMP v2.0.3^33^. Association of taxa to the sample type used for DNA extraction was assessed by running two-sided Welch’s tests on every pair of means. The False Discovery Rate correction^34^ was finally applied and significant differences in taxa between the two experimental conditions were only considered below a p-value of 0.05 and a q-value below 0.2, as in previous works^17^,^35^. Finally, a similarity matrix using the Jaccard index was obtained for all the samples at the family level using PAST v3.0. Similarities between samples were represented in dendrograms built with the Simple Linkage method or with the Neighbour Joining algorithm (using 9,999 repetitions).

## Results and Discussion

During the last few years the growing interest in understanding the human gut microbiota composition has led to a greater knowledge on how these microbial populations may be altered in the framework of certain diseases, notably those with autoimmune or inflammatory components. Gut microbiota is starting to be considered as a dynamic organ of the human body and, as such, susceptible to be transplanted for therapeutic purposes. In all the reported routes and means of administration of FMT the fecal material (fresh or frozen) is diluted in a saline solution, or lyophilized, usually under non-controlled atmospheres.

Separation of microbes from feces using density gradient based methodologies is not a novel concept, as it has been used to separate bacteria from soil since the 1970s. The first separation protocols consisted on repeated blending-centrifugation steps in different buffers and salt solutions^36^,^37^. Later on, bacterial separation by centrifugation on modified sucrose gradients^38^, or by passage through a cation exchange resin (Jacobsen and Rasmussen, 1992) were proposed^39^. The major limitation of those protocols is that they are time consuming and therefore difficult to implement in routine analysis; this limitation is overcome using Nycodenz resine^25^, although no data on bacterial viability is given. In the framework of FMT, separating the GI microbiota from the rest of fecal material offers the advantage of reducing the hygienic concerns due to the unappealing nature of feces.

In this work eight fecal samples from a previous research work addressing the intestinal dysbiosis in the framework of SLE^17^, were chosen. These samples were divergent regarding bacterial diversity as reflected in the different values of the Firmicutes to Bacteroidetes ratio (FBR, lower in SLE patients with respect to healthy controls; HC4 = 8.6, HC32 = 8.8, HC33 = 4.5, SLE2 = 1.6, SLE12 = 1.0, SLE13 = 1.6, SLE21 = 1.2, SLE22 = 0.3). Interestingly, changes in FBR have been observed in certain human disorders such as Crohn’s disease, human type 2 diabetes, or obesity. The rationale underlying this choice was to assess whether our extraction method could interfere in samples with different FBRs.

In our approach, a homogenized fecal dilution was loaded on top of an 80% w/v Nycodenz® solution (Fig. 1A), and centrifuged at 10,000 × g. This differed from the approach of Rooijers *et al.*^40^, which followed the methodology of Murayama *et al.*^41^, with different Nycodenz® gradient preparation and different relative centrifuge force values. Microbiota was separated from the rest of the fecal material in a single centrifugation step. As can be seen in Fig. 1B, two layers corresponding to the microbial biomass were observed in the top of the insoluble debris layer. This debris facilitated the task of microorganism recovery once the upper phase of soluble debris had been removed, as it offered a physical barrier avoiding the mixing of the resuspended microbiota with the lower Nycodenz® layer. The different layers were submitted to contrast phase microscopy, and the vast majority of microorganisms were observed in the above mentioned two layers (Fig. 1C).

The approach employed to evaluate the efficiency of the Nycodenz® extraction procedure showed that the viability of fecal microbiota separated with the density gradient was maintained to a good extent as compared with the fresh fecal microbiota (Suppl. Fig. 1). On average, in the microbiota recovered after the Nycodenz® treatment 66.9% of the bacteria were still alive, with values ranging between 71.3 and 60.6% (66.9 ± 5.6) among the three fecal samples analyzed, meanwhile in fresh feces the viability was estimated to range between 85.6–49.9% (68.6 ± 17.9). This gives an idea of the good efficiency of the methodology proposed for the concentration and isolation of viable microbiota, regardless the variation of feces in terms of humidity and fiber content. The concentrations of live bacteria in the three samples analyzed by flow cytometry ranged between 3.2 × 10^9^ and 7.2 × 10^9^ (5.2 ± 2.0 × 10^9^) bacteria/gram of fecal dry matter in the original samples of fresh feces, and between 5.7 × 10^9^ and 9.0 × 10^9^ (7.0 ± 1.8 × 10^9^) per gram of feces after density gradient extraction. This means that all the viable bacteria are extracted from the fecal material, with a yield of around 10^10^ viable bacteria per two grams of fecal sample. No significant differences were found in mean concentrations before and after the treatment. Thus, our results showed minimal variability in the viable microbiota recovered among different individual donors, and no impact of the isolation protocol over the integrity of the fecal bacteria.

**Figure 2:**
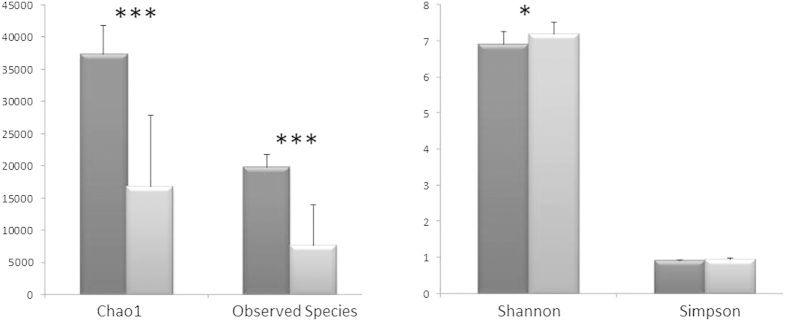
Different alpha diversity indices obtained from the stool samples before (dark gray) or after (light gray) microbiota separation by density gradient. Bars represent the Mean ± Standard Deviation. (*p < 0.05; ***p < 0.001).

The analysis of the microbiota fractions extracted using the density gradient resulted in an overall decrease of diversity when compared to the results obtained from the DNA extracted directly from feces. This was true when alpha-diversity indices that take into account only species richness (Chao 1, Observed Species) were calculated, but the contrary was observed when using the Shannon index, which takes into account species evenness (Shannon) (Fig. 2). No difference in alpha-diversity was detected using the Simpson index. Overall, this means that although the number of recovered OTUs is lower after Nycodenz® extraction, the proportions between them did not necessarily vary during the extraction process. Care should be taken in the sense that reductions in alpha-diversity might affect FMT effectiveness, as precise bacterial groups important to the balance of dysbiosis could be lost. In this way, further research is needed to determine if the reduction in OTUs/species could be in part due to oxygen exposure during manipulation of the microbiota separation in the density gradient. It is possible that particular microbial types, more susceptible to oxygen, may be protected if the fecal microbiota extraction is performed under strict anaerobic conditions. It will be also interesting to elucidate if Nycodenz® gradients are helpful in selectively removing undesirable molecules/microorganisms from the feces, such as toxins, prions and viruses.

**Figure 3:**
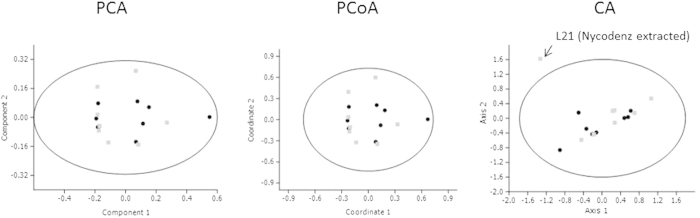
Different non-biased multivariate ordering methodologies were used in order to determine whether the microbial populations obtained directly from homogenized stool samples (black dots), or from the separated fecal microbiota (gray squares) clustered apart according to their composition. Ellipses represent the estimated region where 95% of population points were expected to fall. Analyses were performed at the Phylum and Family levels. PCA: Principal Component Analysis; PCoA, Principal Coordinates Analysis; CA, Correspondence Analysis.

In order to know whether the differences observed in microbial diversity between the samples, with or without Nycodenz® extraction, could be used for clustering purposes, samples were ordered using the taxonomic composition at the Phylum or Family level through different methodologies: Principal Components Analysis (PCA), Principal Coordinates Analysis (PCoA) and Correspondence Analysis (CA) (Fig. 3). When used in an unbiased way, i.e. without providing information on the source of the different microbial profiles, all of the ordering methods were able to cluster samples into separated groups (feces vs extracted microbiota) (Fig. 3). Absence of effect of the extraction method was sustained statistically, *a posteriori*, with the use of non-parametric tests such as One-way ANOSIM and One-way PERMANOVA. In both cases, samples were firstly classified according to their origin and their similarities measured according to Euclidean distances. The p-values obtained did not support the classification of samples into two groups (feces vs extracted microbiota; One-way ANOSIM; p-value 0.733; One-way PERMANOVA; p-value 0.353).

**Figure 4:**
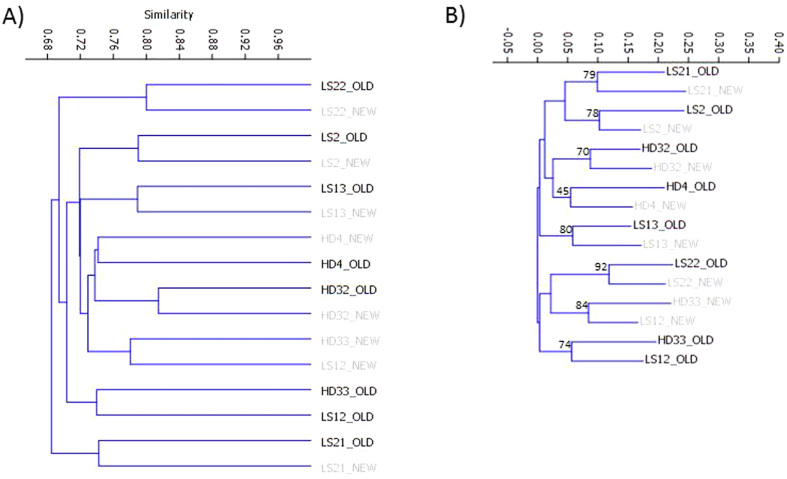
Dendrograms showing similarity of samples according to Jaccard distances; (**A**) clustering through simple linkage; (**B**) clustering using to Neigbour Joining with branch support (10,000 repetitions). NEW and OLD suffixes denote samples where microbiota was or was not extracted in the density gradient, respectively, prior to DNA extraction.

In order to further study the effect of microbial separation by density gradient on the 16S RNA gene profiling, a similarity matrix using the relative family abundances was built by calculating Jaccard distances, a method already used in other metagenomic studies^42^. Samples were clustered using those inter-sample distances according to the Simple Linkage method, or through the Neighbour Joining algorithm, and the corresponding dendrograms obtained (Fig. 4). In both cases, samples in which the DNA was extracted after microbiota separation, clustered with their corresponding feces samples, with the exception of samples LS12 and HD33. In these samples, the effect of the microbiota extraction over the metagenomic profiles was higher, with some groups showing drastic changes at the phylum or family levels (Suppl. Fig. 2). These results confirmed that, as a general rule, the microbial communities extracted using the density gradient centrifugation procedure are representative of those present in the original stool sample.

Separation of microorganisms using a Nicodenz® density gradient was first introduced for the isolation of bacteria from soil by Lindahl and Bakken^43^. This method has also been successfully applied in other biological systems, such as in the description of the intestinal metagenome of the red palm weevil (*Rhynchophorus ferrugineus*)^44^, or in the gene expression assessment in dairy matrices^45^. Separating bacteria from certain matrix compounds may be very useful for downstream molecular biology applications, as this step removes many of the components inhibiting PCR, such as humic compounds, or colored substances interfering with blot hybridization protocols^46^.

In our work, the application of this methodology was shown to not affect the global variability of the extracted microbiota, and the diversity of bacteria extracted directly from the soil or following Nycodenz® gradient was not significantly different, with the exception of γ-Proteobacteria^25^. However, some taxonomic groups showed significant variations according to the gradient extraction when the totality of the samples was grouped and analyzed (Table 1). In general, 16S rRNA gene profiles from samples in which microbiota was extracted in the Nycodenz® gradient were characterized for higher relative abundances of the Firmicutes phylum, this being due to higher recoveries of the Clostridiales order (Suppl. Fig. 3). Several groups form the Beta, Delta, and Epsilon divisions of the Proteobacteria phyla also showed significant variations according to treatment, although to a lesser extent.

In general, the methodology presented in this work offers a simple and straight-forward method to extract and separate the fecal microbiota from the rest of stool components, allowing further improvements such as performing this process under controlled atmospheric conditions. In addition, this extraction step may eliminate some undesirable compounds of the feces, but this deserves further research. Our approach could be of use in obtaining representative intestinal microbiota free of stool material for long-term storage purposes. This might also be helpful as the first step for preserving the overall microbial communities, and used in the near future in the design of microbiota-based products/vehicles for FMT and novel intestinal restoration bio-therapies. However, further improvements of this method are needed as, for instance, some *Clostridium*-related OTUs were significantly affected by the Nycodenz® extraction, and some members of this genus such as *C. scindens* can be relevant in the CDI treatment^47^. In addition, animal experiments are needed in order to show that microbiota extracted following this method is effectively engrafted in the host.

Interestingly, this protocol can be scaled up allowing the processing of larger fecal amounts with centrifugal devices with higher capacity. For instance, 430 grams of fecal material can be processed using swinging rotors (up to 30,000 × *g*) allocating 4 × 1000 mL buckets, with an expected yield of 10^12^ viable bacteria.

To sum up, obtaining the representative microbiota from the feces of a healthy donor using the Nycodenz® density gradient described in this work would allow the concentration of intestinal microbiota and keep it separated from the rest of the stool components, whilst maintaining high viability levels. On one hand, Nycodenz® is a safe molecule, in terms of human toxicity, which can easily be removed from samples in the process of microbiota extraction. With respect to other molecules used for density gradient isolation, the X-ray dense-compound Nycodenz® shows advantages, such as a non-inhibitory effect on the activity of most enzymes, compatibility to protein determination assays and, what is relevant for the purpose of this paper, it shows a low toxicity in human beings^41^. On the other hand, density gradient allows for the recovery of representative and viable fecal microbiota and the suppression of non-desirable microorganisms/compounds, as well as facilitating the testing of the samples for hazardous agents. This might allow the development of downstream applications such as microbiota-based therapeutic strategies for microbial intestinal restoration, both in the framework of a given disease or for other applications.

## Additional Information

**How to cite this article**: Hevia, A. *et al.* Application of density gradient for the isolation of the fecal microbial stool component and the potential use thereof. *Sci. Rep.* **5**, 16807; doi: 10.1038/srep16807 (2015).

## Supplementary Material

Supplementary Information

## Figures and Tables

**Table 1:** Taxons showing statistical differences in their relative abundances when comparing direct DNA extraction from faeces or after density gradient separation of the microbiota.

Taxon	Faeces Mean^a^ ± SD^b^	Gradient Mean^c^	SD^d^	p^e^	FDR^f^
Phylum					
Firmicutes	58.98 ± 16.17	78.94	13.82	0.02	0.14
Proteobacteria	1.87 ± 1.32	0.18	0.19	0.01	0.11
Class					
Clostridia	57.87 ± 16.00	77.43	13.49	0.02	0.20
Erysipelotrichi	0.29 ± 0.20	0.05	0.03	0.01	0.16
Betaproteobacteria	0.78 ± 0.86	0.01	0.01	0.04	0.17
Deltaproteobacteria	0.13 ± 0.07	0.07	0.04	0.04	0.16
Epsilonproteobacteria	0.00^g^ ± 0.00	0.00	0.00	0.02	0.19
Unclassified Proteobacteria Class	0.01 ± 0.01	0.00	0.00	0.03	0.18
Opitutae	0.18 ± 0.20	0.00	0.00	0.03	0.19
Order					
Unclassified Bacteroidetes Order	0.05 ± 0.02	0.03	0.02	0.01	0.01
Clostridiales	57.60 ± 15.90	77.12	13.44	0.02	0.02
Erysipelotrichales	0.29 ± 0.20	0.05	0.03	0.01	0.01
Burkholderiales	0.76 ± 0.84	0.01	0.01	0.04	0.04
Desulfovibrionales	0.13 ± 0.07	0.06	0.04	0.04	0.04
Unclassified Proteobacteria Order	0.01 ± 0.01	0.00	0.00	0.03	0.03
Cerasicoccales	0.16 ± 0.17	0.00	0.00	0.03	0.03
Family					
Odoribacteraceae	0.25 ± 0.11	0.05	0.04	0.00	0.10
Unclassified Clostridiales Family	3.35 ± 1.44	6.57	2.10	0.00	0.16

^a^Original: mean rel. freq. (%).

^b^Original: std. dev. (%).

^c^Gradient extracted: mean rel. freq. (%).

^d^Gradient extracted: std. dev. (%).

^e^P-values.

^f^False Discovery Rate (30).

^g^values > 0.001.
