# Supplementary material for: Application of density gradient for the isolation of the fecal microbial stool component and the potential use thereof
Source: Sci Rep. 2015 Nov 19;5:16807. doi: 10.1038/srep16807 (PMC4652190; doi:10.1038/srep16807)
Supplement: Supplementary Information [file srep16807-s1.pdf]

SUPPLEMENTARY INFORMATION

Title: Application of density gradient for the isolation of the fecal microbial stool component and the potential use thereof.

Authors list: Arancha Hevia, Susana Delgado, Abelardo Margolles, Borja Sánchez

Supplementary Figure 1

A.1) Fecal microbiota before Nycodenz® separation

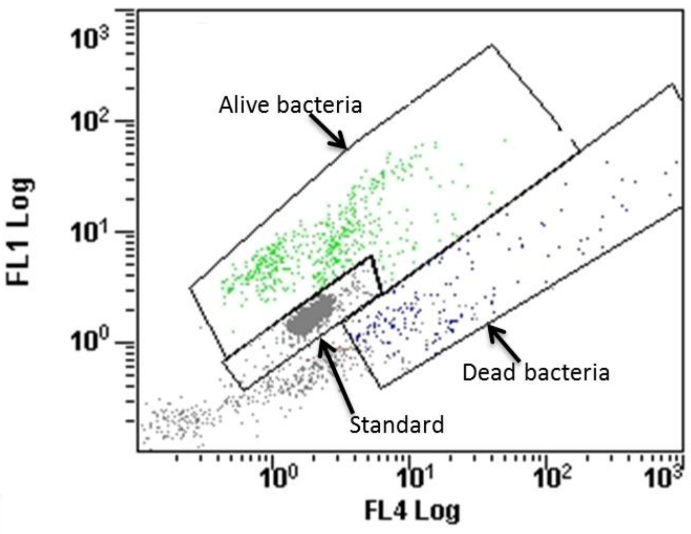

B.1) Fecal microbiota after separation

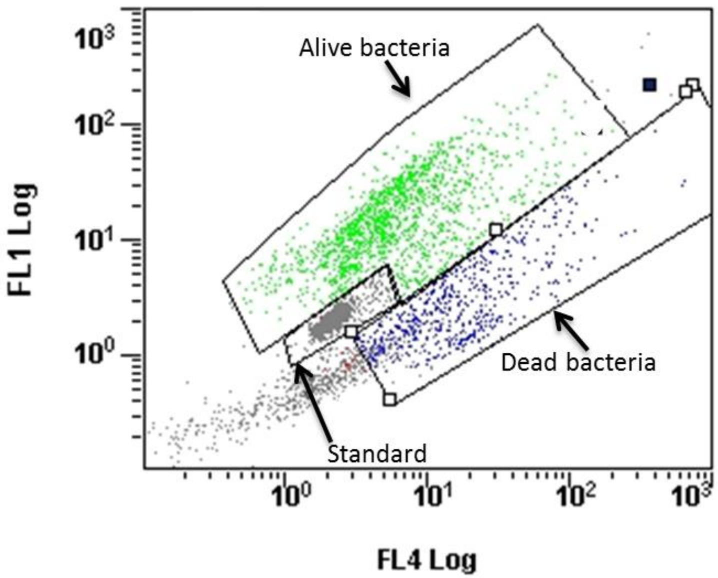

A.2) Control of dead microbiota before separation.

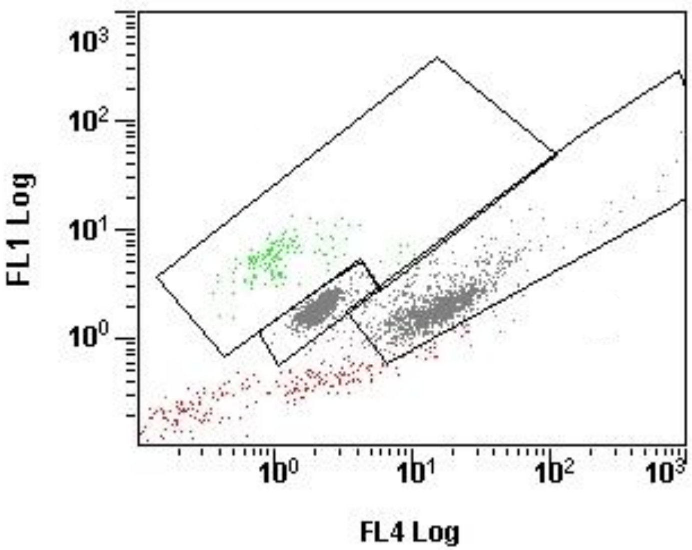

B.2) Control of dead microbiota after separation

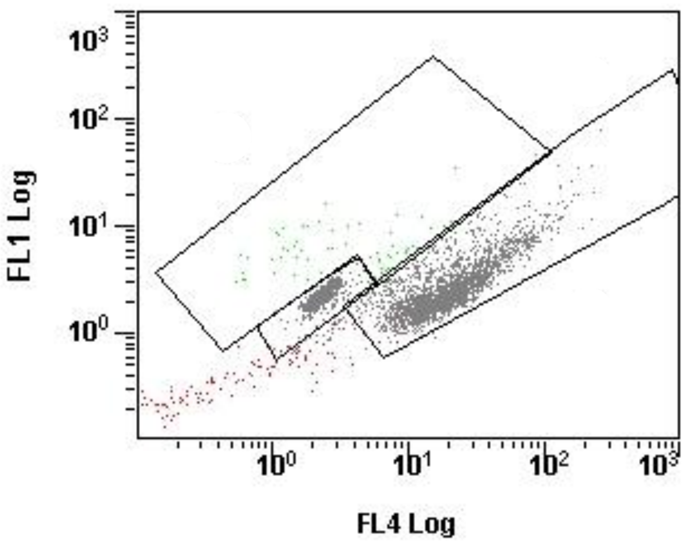

Supplementary Figure 1

Suppl. Figure 1. Flow cytometry diagrams showing the proportions of alive and dead bacteria in a representative sample before and after Nycodenz extraction. The two diagrams on the bottom represent control samples in which all bacteria were killed.

Supplementary Figure 2

Phylum level

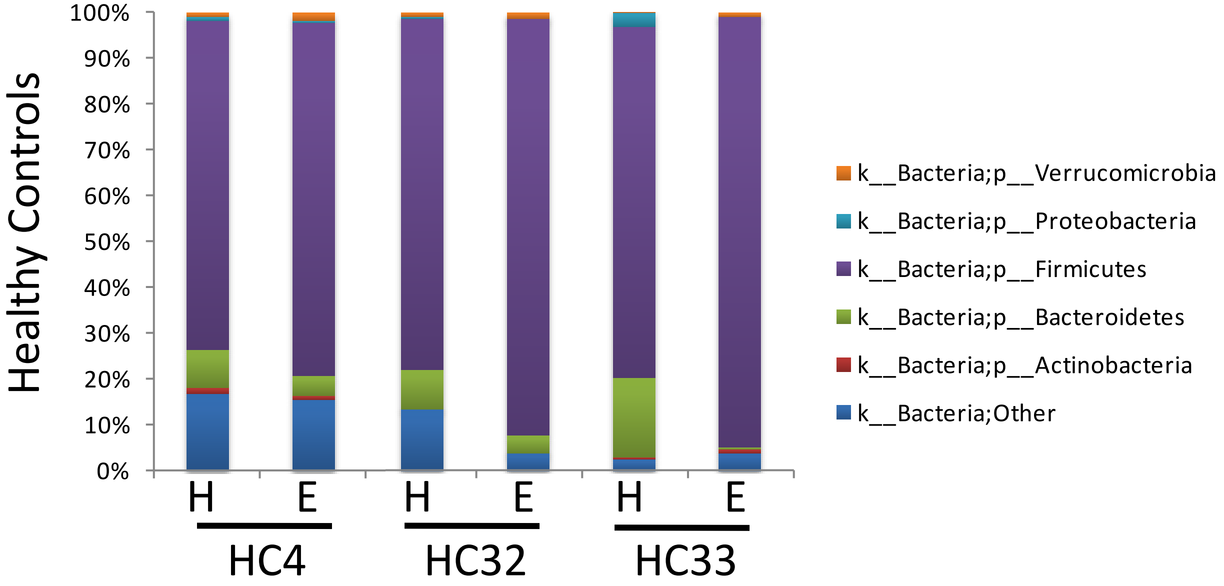

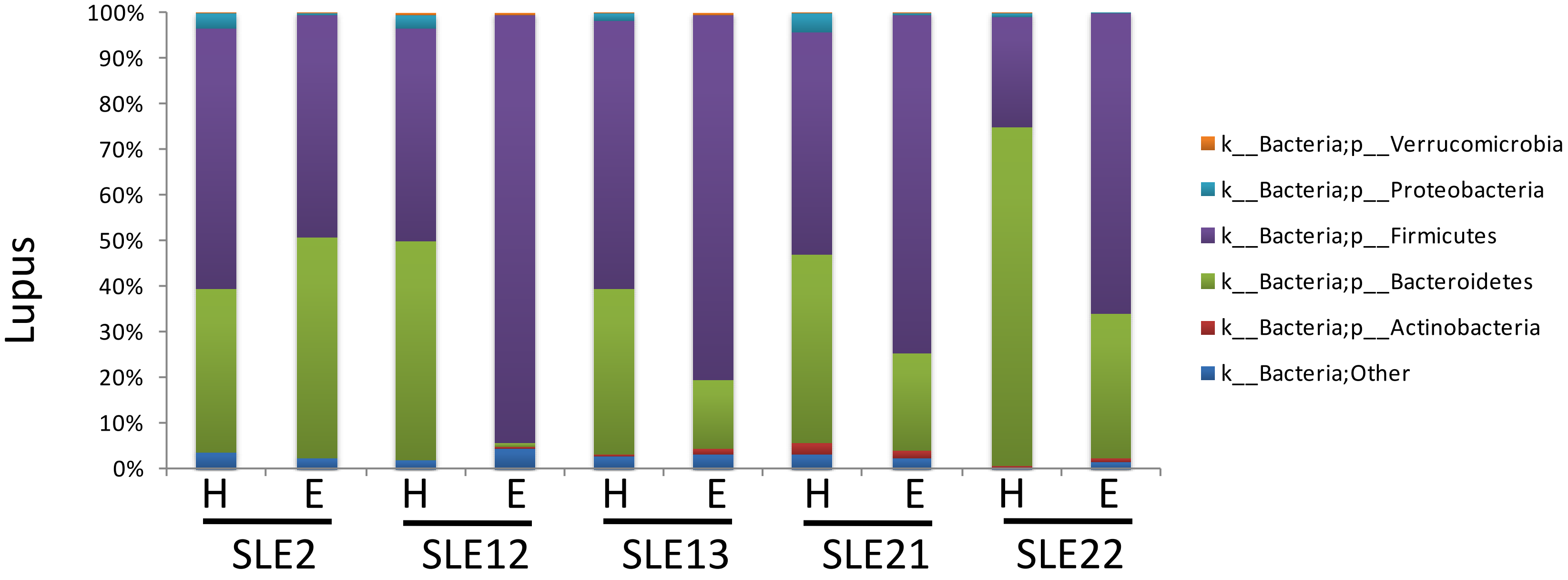

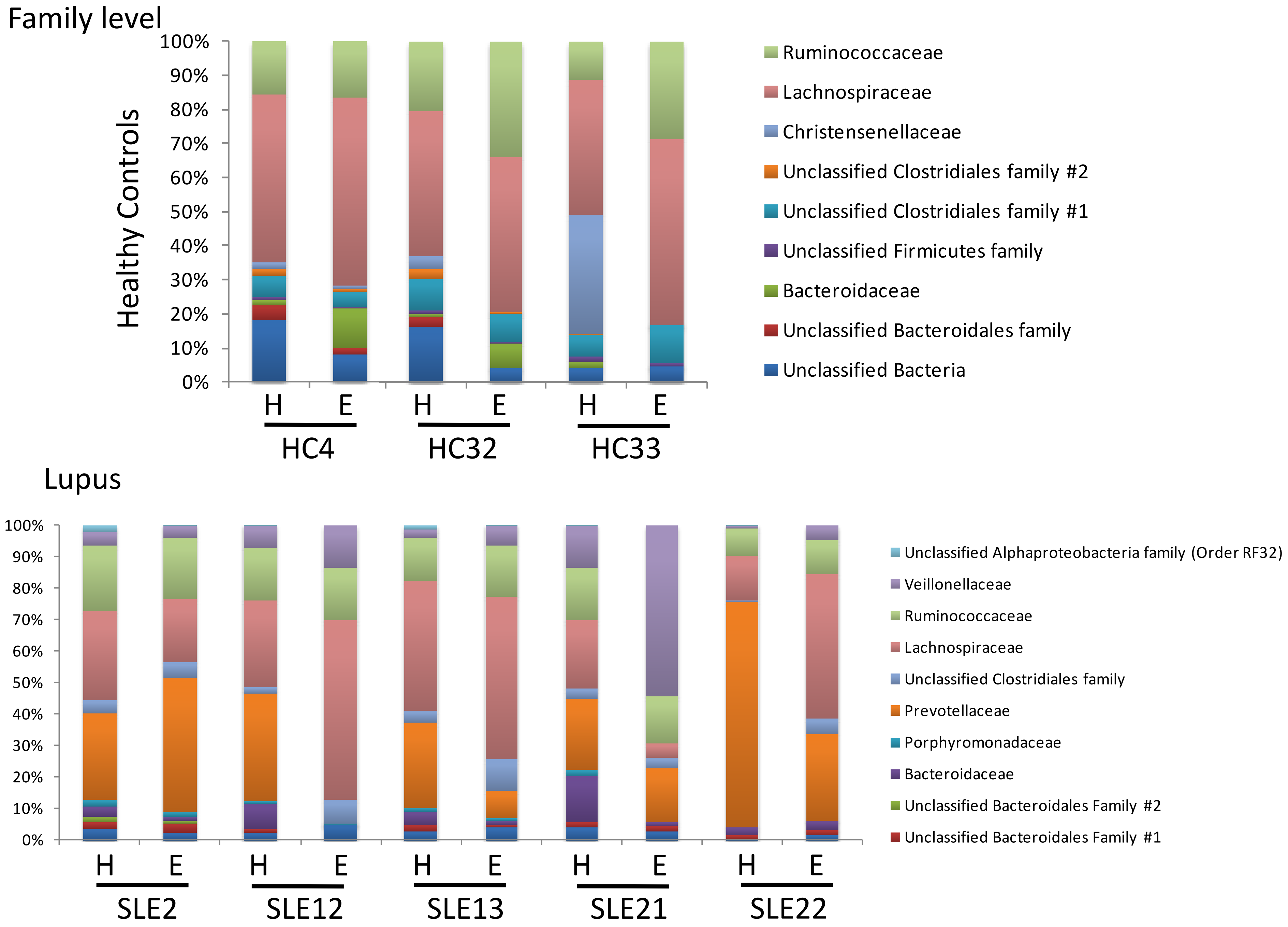
Family level
Healthy Controls
100%
90%
80%
70%
60%
50%
40%
30%
20%
10%
0%
H
E
HC4
HC32
HC33
Ruminococcaceae
Lachnospiraceae
Christensenellaceae
Unclassified Clostridiales family #2
Unclassified Clostridiales family #1
Unclassified Firmicutes family
Bacteroidaceae
Unclassified Bacteroidales family
Unclassified Bacteria
Lupus
SLE2
SLE12
SLE13
SLE21
SLE22
Unclassified Alphaproteobacteria family (Order RF32)
Veillonellaceae
Ruminococcaceae
Lachnospiraceae
Unclassified Clostridiales family
Prevotellaceae
Porphyromonadaceae
Bacteroidaceae
Unclassified Bacteroidales Family #2
Unclassified Bacteroidales Family #1

Supplementary Figure 2

Suppl. Figure 2. Microbial composition of the different samples used in this study at the Phylum and Family levels, represented as the relative sequence abundances (%). Paired samples (H, homogenized stool sample vs E, microbiota extracted sample) are grouped in Systemic Lupus Erythematosus (SLE) patients and Healthy Controls (HC).

Suppl. Figure 3

Phylum

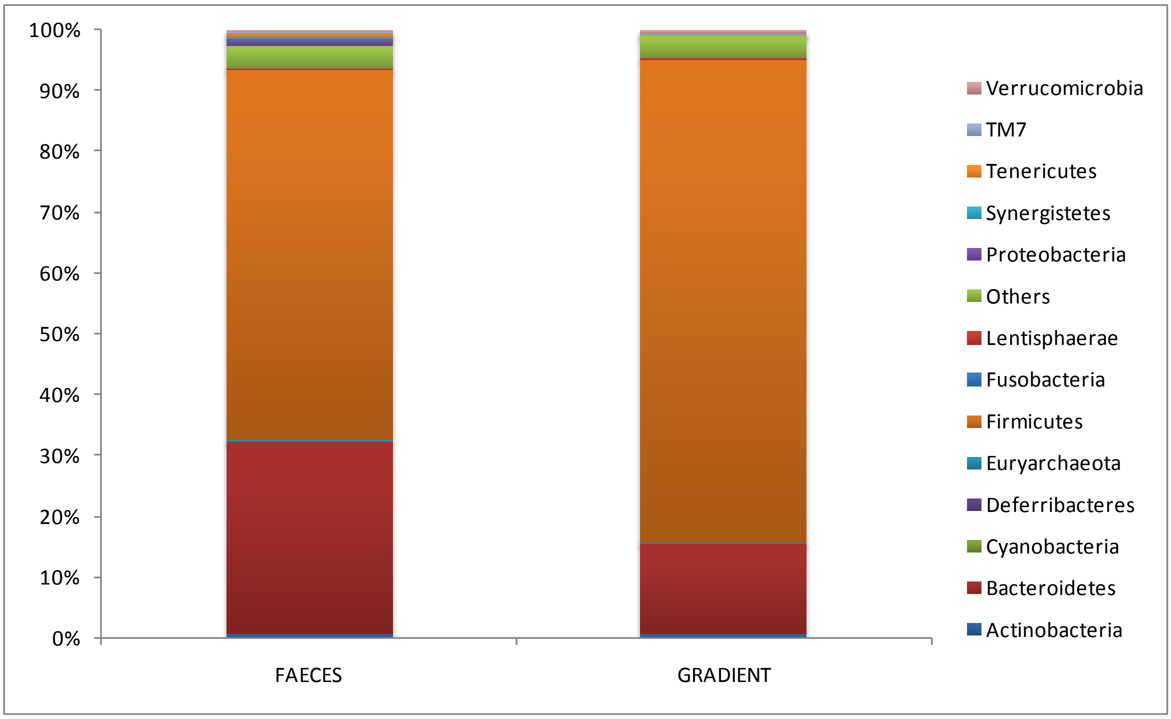

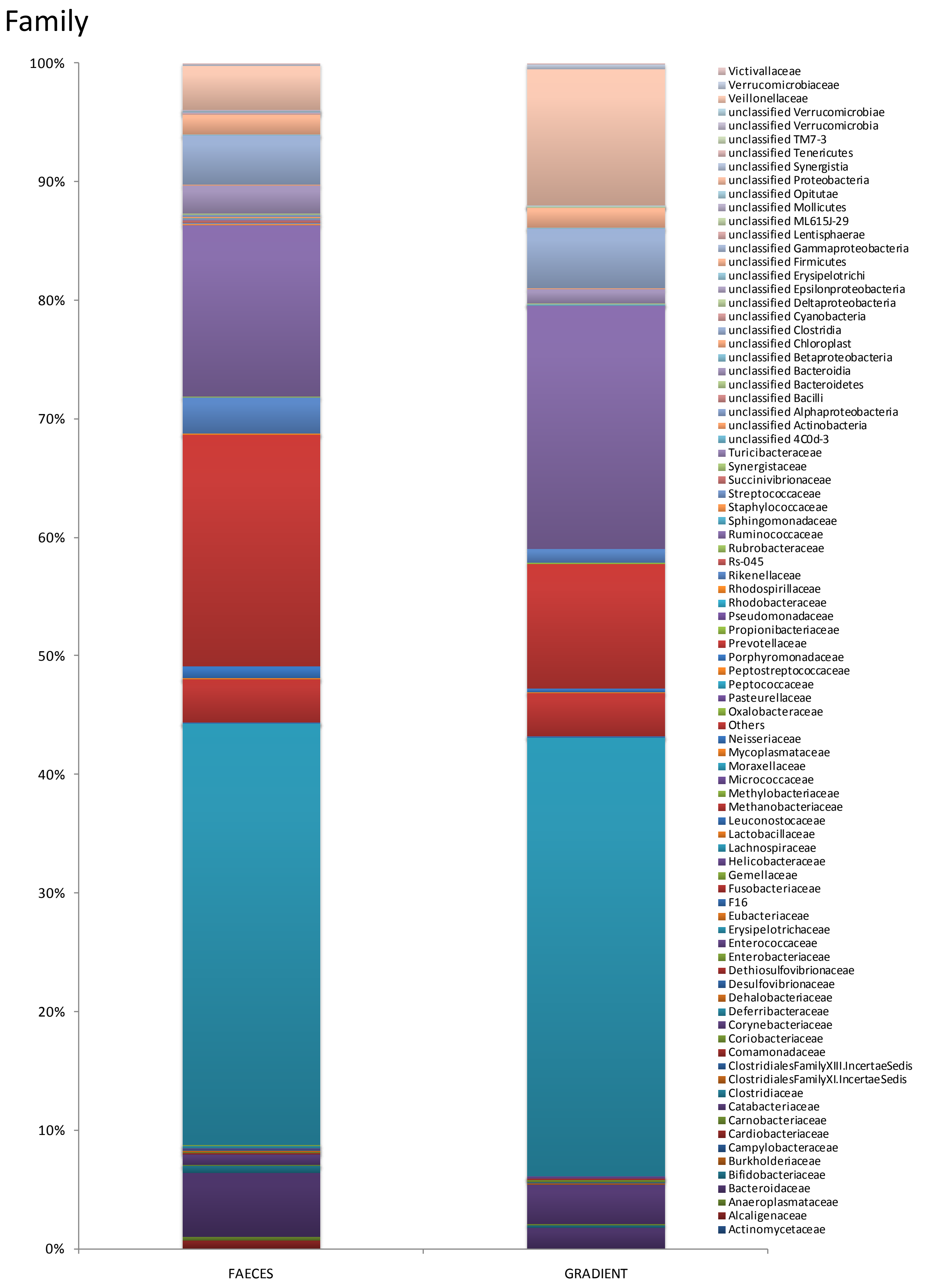
Family
100%
90%
80%
70%
60%
50%
40%
30%
20%
10%
0%
FAECES
GRADIENT
Victivallaceae
Verrucomicrobiaceae
Veillonellaceae
unclassified Verrucomicrobiae
unclassified Verrucomicrobia
unclassified TM7-3
unclassified Tenericutes
unclassified Synergistia
unclassified Proteobacteria
unclassified Opitutae
unclassified Mollicutes
unclassified ML615J-29
unclassified Lentisphaerae
unclassified Gammaproteobacteria
unclassified Firmicutes
unclassified Erysipelotrichi
unclassified Epsilonproteobacteria
unclassified Deltaproteobacteria
unclassified Cyanobacteria
unclassified Clostridia
unclassified Chloroplast
unclassified Betaproteobacteria
unclassified Bacteroidia
unclassified Bacteroidetes
unclassified Bacilli
unclassified Alphaproteobacteria
unclassified Actinobacteria
unclassified 4C0d-3
Turicibacteraceae
Synergistaceae
Succinivibrionaceae
Streptococcaceae
Staphylococcaceae
Sphingomonadaceae
Ruminococcaceae
Rubrobacteraceae
Rs-045
Rikenellaceae
Rhodospirillaceae
Rhodobacteraceae
Pseudomonadaceae
Propionibacteriaceae
Prevotellaceae
Porphyromonadaceae
Peptostreptococcaceae
Peptococcaceae
Pasteurellaceae
Oxalobacteraceae
Others
Neisseriaceae
Mycoplasmataceae
Moraxellaceae
Micrococcaceae
Methylobacteriaceae
Methanobacteriaceae
Leuconostocaceae
Lactobacillaceae
Lachnospiraceae
Helicobacteraceae
Gemellaceae
Fusobacteriaceae
F16
Eubacteriaceae
Erysipelotrichaceae
Enterococcaceae
Enterobacteriaceae
Dethiosulfovibrionaceae
Desulfovibrionaceae
Dehalobacteriaceae
Deferribacteraceae
Corynebacteriaceae
Coriobacteriaceae
Comamonadaceae
ClostridialesFamilyXIII.IncertaeSedis
ClostridialesFamilyXI.IncertaeSedis
Clostridiaceae
Catabacteriaceae
Carnobacteriaceae
Cardiobacteriaceae
Campylobacteraceae
Burkholderiaceae
Bifidobacteriaceae
Bacteroidaceae
Anaeroplasmataceae
Alcaligenaceae
Actinomycetaceae

Suppl. Figure 3

Suppl. Figure 3. Cumulative microbial composition of the samples grouped by experimental condition (DNA extracted directly from feces of after density gradient extraction). Graphics were plotted at the Phylum and Family levels, and data is represented as relative abundances (%). In the case of the Phylum level, an additional graphic excluding the two more abundant taxa (Bacteroidetes and Firmicutes) is shown in order to evaluate changes in minority phyla.
